# Safety and efficacy of long-term combination therapy with bezafibrate and ezetimibe in patients with dyslipidemia in the prospective, observational J-COMPATIBLE study

**DOI:** 10.1186/1475-2840-12-163

**Published:** 2013-11-06

**Authors:** Tamio Teramoto, Kazunori Abe, Takehiko Taneyama

**Affiliations:** 1 Teikyo Academic Research Center, Teikyo University, 2-11-1, Kaga, Itabashi-ku, Tokyo 173-8606, Japan; 2 Pharmacovigilance & Post-marketing Surveillance, KISSEI Pharmaceutical Co., Ltd, 3-1-3, koishikawa, Bunkyou-ku, Tokyo 112-0002, Japan

**Keywords:** Bezafibrate, Ezetimibe, Combination therapy, Gallstone, Low-density lipoprotein cholesterol, High-density lipoprotein cholesterol, Triglyceride, Non-high-density lipoprotein cholesterol, Dyslipidemia

## Abstract

**Background:**

There are numerous reports describing the efficacy of fenofibrate in combination with ezetimibe for treating dyslipidemia. In contrast, a study combining bezafibrate and ezetimibe has not yet been conducted. In this study, we examined the safety, including the risk of gallstone formation, and the efficacy of long-term combination therapy with bezafibrate and ezetimibe for treating dyslipidemia.

**Methods:**

Dyslipidemic patients treated with 400 mg/day bezafibrate in combination with 10 mg/day ezetimibe for the first time were eligible. We selected 157 institutions in Japan and conducted a 12-month prospective observational study, with patients enrolled on the day they started combination therapy. Safety of the combination was examined in terms of the type, onset, and severity of adverse drug reactions (ADRs). Efficacy was evaluated in terms of the changes in low-density lipoprotein-cholesterol (LDL-C), high-density lipoprotein-cholesterol (HDL-C), triglyceride (TG), and non-HDL cholesterol (non-HDL-C) levels from the start of combination therapy (baseline) to the last observation carried forward (LOCF). Lipid levels were assessed at 1, 3, 6, and 12 months after starting combination therapy.

**Results:**

We enrolled 665 patients in this observational study. Safety was evaluated in 659, and ADRs occurred in 42 patients (6.4%). The most frequent ADRs were blood creatine phosphokinase increase (1.5%) and myalgia (0.8%). Asymptomatic gallstones were observed in four patients (0.6%). Effectiveness was evaluated in 622 patients. LDL-C, HDL-C, TG, and non-HDL-C levels improved significantly from baseline to LOCF by −17.4%, 8.8%, –40.5%, and −21.6%, respectively (all, p < 0.001). Lipid levels also improved from baseline to each evaluation time-point.

**Conclusions:**

Bezafibrate in combination with ezetimibe is safe and effective, and is potentially useful for comprehensive management of dyslipidemia.

## Background

Low-density lipoprotein-cholesterol (LDL-C) is one of the most important factors for evaluating the risk associated with cardiovascular disorders. Statins are the most commonly used drug for treating elevated LDL-C, and reduce the risk of cardiovascular events, as demonstrated in large-scale clinical trials [1].

Although statin therapy reduces the risk of cardiovascular events by approximately 30%, the residual risk of cardiovascular events is approximately 70% [2]. Therefore, it is necessary to consider additional interventions to reduce the residual risk. In addition to LDL-C, low levels of high-density lipoprotein-cholesterol (HDL-C) and high triglyceride (TG) concentrations are considered to be risk factors for coronary artery disease [3-5]. For this reason, it is increasingly being acknowledged that integrated management of dyslipidemia targeting LDL-C as well as HDL-C and TG is necessary [6,7]. In particular, the European Society of Cardiology/the European Atherosclerosis Society [8] and the Japan Atherosclerosis Society [9] recommend fibrates as the Class I pharmaceutical therapy for treating hypertriglyceridemia.

Fibrates activate peroxisome proliferator-activated receptor (PPAR) alpha, a nuclear receptor that affects the transcriptional regulation of many genes governing lipoprotein metabolism, thereby decreasing TG levels while increasing HDL-C levels [10]. One of these drugs, bezafibrate, is a ligand for three PPAR subtypes (alpha, gamma, and delta) and exerts unique actions that differ from those of other fibrates [10,11]. In contrast, ezetimibe is an inhibitor of Niemann-pick C1 like 1 protein (NPC1L1), an intestinal cholesterol transporter that is localized to the small intestinal mucosa. By inhibiting NPC1LI, ezetimibe reduces lipid uptake from the intestine, and thereby reduces hepatic cholesterol content and circulating LDL-C levels [12].

Because of these independent mechanisms of action, a combination of bezafibrate and ezetimibe is expected to provide a comprehensive treatment option for dyslipidemia by decreasing LDL-C, increasing HDL-C, and markedly decreasing TG levels. In fact, several studies have already documented the efficacy of fenofibrate in combination with ezetimibe [13-15]. In contrast, a study combining bezafibrate and ezetimibe has not yet been conducted. For this reason, we conducted a 12-month prospective observational study (J-COMPATIBLE study; Japanese safety and efficacy of long-term COMbination theraPy with bazafibrAte and ezeTImiBe in patients with dysLipidEmia study) the safety and efficacy of bezafibrate in combination with ezetimibe for the treatment of dyslipidemia.

## Methods

### Subjects

This prospective observational study was conducted between January 2009 and February 2011 and involved 157 medical institutions in Japan. After obtaining written informed consent, a central registration system was used to enroll patients who satisfied all eligibility criteria. This study was conducted in accordance with Good Post-marketing Study Practice (GPSP) of the Ministry of Health, Labour, and Welfare, Japan. Approval by the ethics committee of each institution was not mandatory, because GPSP does not require such approval for Post-Marketing Surveillance.

Dyslipidemic patients who received combination therapy with bezafibrate and ezetimibe for the first time were eligible for enrollment. Patients with any of the following were excluded: (1) gallstone, suspected gallstone, or history of gallstone; (2) blood creatinine ≥ 2.0 mg/dL within 1 month before starting treatment; (3) severe renal disease (defined as on dialysis or renal failure); or (4) severe liver disease.

### Study design

Before starting the study, patients treated with 400 mg/day bezafibrate added 10 mg/day ezetimibe to their ongoing bezafibrate regimen, or vice versa. Patients who were naïve to both drugs began taking these two drugs concurrently at the start of the study. The study period was defined as that from the start of combination therapy (baseline) to 12 months of treatment or until patients discontinued the regimen.

Safety was evaluated in all patients except those with protocol violations or for whom there were insufficient data for assessing safety. Efficacy was evaluated for all patients except those who did not comply with drug administration, had protocol violations, or who had insufficient data for assessing safety.

### Safety assessments

We evaluated adverse drug reactions (ADRs) during combination therapy with bezafibrate and ezetimibe. Patients underwent ultrasonography at baseline and at the end of the observation period to detect the presence of gallstones. Safety parameters included body mass index (BMI), total bilirubin, blood creatinine, blood urea nitrogen, aspartate aminotransferase (AST), alanine aminotransferase (ALT), gamma-glutamyl transpeptidase (GGT), alkaline phosphatase (ALP), creatine phosphokinase (CPK), systolic blood pressure, diastolic blood pressure, and glycated hemoglobin. Glycated hemoglobin was measured according to the Japan Diabetes Society method [16] and values were converted to National Glycohemoglobin Standardization Program values.

### Efficacy assessments

Efficacy parameters were LDL-C, HDL-C, TG, and non-HDL cholesterol (non-HDL-C). These values were measured at baseline, and after 1, 3, 6, and 12 months of treatment. LDL-C was calculated using the Friedewald formula (LDL-C = total cholesterol – HDL-C – TG/5), in patients with TG < 400 mg/dL [17]. Non-HDL-C was calculated by subtracting HDL-C from total cholesterol (data not shown).

### Statistical analysis

All statistical analyses were performed using SAS version 9.2 (SAS Institute Inc., Cary, NC, USA). Data are shown as means ± standard deviation (SD). The cumulative incidence of ADRs was estimated using the Kaplan–Meier method. The changes from baseline to the last observation carried forward (LOCF), or to specific time-points, in safety and efficacy parameters were analyzed using a two-tailed paired *t*-test. Furthermore, all patients were classified into three groups according to drug administration background at the start of the study: (1) patients who were already taking ezetimibe and then added bezafibrate (EZE + BEZA group); (2) patients who were already taking bezafibrate and then added ezetimibe (BEZA + EZE group); and (3) patients who started bezafibrate and ezetimibe concurrently (BEZA & EZE group). For inter-group comparisons of ADRs, the *χ*^2^ test was used for statistical analysis. For lipid parameters, the differences among the three groups were evaluated by analysis of covariance (ANCOVA), with application of the model to the baseline value as the covariate and the change in each efficacy variable. The statistical significance level was set at 5%. Patients without ADRs were included in the analysis with the day they stopped combination therapy as the treatment termination point. The LOCF method was used to impute missing data.

The necessary sample size was calculated to be ≥ 500 patients based on two studies of fenofibrate in combination with ezetimibe, in which Farnier et al. enrolled 625 patients [13] and McKenney et al. enrolled 576 patients [14].

## Results

### Patients

Of 665 patients enrolled in the study, safety was evaluated in 659 (Table 1) after excluding five patients in whom ADRs could not be identified and one patient who did not receive combination therapy. The mean age ± SD of the patients was 60.8 ± 13.1 years. Concurrent diseases were observed in 83.6% of patients, and included hypertension (55.8%), diabetes (34.9%), hepatic disease (23.1%), cardiac disease (7.0%), and renal disease (3.0%). In 91.7% of patients, combination therapy was planned as a primary prevention based on the Japan Atherosclerosis Society Guidelines [9]. Fourteen patients (2.1%) also received statins.

**Table 1:** Baseline patient characteristics (safety analysis set)

**Characteristics**	**n**	**mean ± SD/%**
Age (years)	659	60.8 ± 13.1
Men (%)	384	58.3
With complications (%)	551	83.6
Concurrent disease		
Hypertension (%)	368	55.8
Diabetes (%)	230	34.9
Hepatic disease (%)	152	23.1
Cardiac disease (%)	46	7.0
Renal disease (%)	20	3.0
Purpose of combination therapy ^a^		
Primary prevention (%)	604	91.7
Secondary prevention (%)	26	3.9
Unknown (%)	29	4.4
Concomitant medications		
Drugs for hypertension (%)	355	53.9
Drugs for diabetes (%)	163	24.7
Statin (%)	14	2.1

Data are expressed as mean ± standard deviation or n and percent.

^a^Defined according to the Guidelines for the Prevention of Atherosclerotic Cardiovascular Disease 2007 [9].

Efficacy was evaluated in 622 patients, after excluding the six patients who had been excluded from the safety assessment, 10 who had a history of gallstones, 13 with registration violations, and 14 who did not comply with the drug administration regimen.

### Safety assessments

Of the 659 patients included in the safety analysis, 42 (6.4%) experienced ADRs (Table 2). The most common ADRs were blood CPK increase (1.5%), myalgia (0.8%), gallstone (0.6%), increased blood creatinine (0.6%), and increased AST (0.5%). Severe ADRs occurred in three patients, and included increases in both blood CPK and blood creatinine in one patient, gastric cancer in one patient, and both bleeding stomach ulcer and gastric cancer in one patient. These three patients discontinued combination therapy, and all recovered with appropriate treatment. The cumulative incidences of ADRs were 4.0% (95% confidence interval [CI]: 2.7–5.8%), 4.8% (95% CI: 3.4–6.8%), and 6.2% (95% CI: 4.6–8.4%) at 3, 6, and 12 months, respectively, with no marked increase in the incidence of ADRs over time. Of 359 patients who underwent ultrasonography at baseline and at the completion of observation, four had asymptomatic gallstones at the end of the observation period.

**Table 2:** Adverse drug reactions in the safety analysis set

**Adverse drug reactions**	**n**	**%**
Patients evaluated	659	−
Patients with ADRs	42	6.4
ADRs in ≥ 0.3% of patients		
Blood CPK increased	10	1.5
Myalgia	5	0.8
Gallstone	4	0.6
Blood creatinine increased	4	0.6
AST increased	3	0.5
Gastric cancer	2	0.3
Diabetes	2	0.3
Renal dysfunction	2	0.3
ALT increased	2	0.3
Blood TG increased	2	0.3

ADRs: adverse drug reactions; CPK: creatine phosphokinase; AST: aspartate aminotransferase; ALT: alanine aminotransferase; TG: triglyceride.

The changes in safety parameters are presented in Table 3. Total bilirubin, AST, ALT, GGT, ALP, systolic blood pressure, and diastolic blood pressure decreased significantly from baseline to the LOCF, while creatinine and blood urea nitrogen increased significantly.

**Table 3:** Changes in safety parameters

**Parameter**	**n**	**Baseline**	**LOCF**	**p-value**
Body mass index (kg/m^2^)	362	25.49 ± 3.72	25.40 ± 3.77	0.087
Total bilirubin (mg/dL)	292	0.64 ± 0.25	0.58 ± 0.22	<0.001
Creatinine (mg/dL)	449	0.76 ± 0.17	0.78 ± 0.19	<0.001
Blood urea nitrogen (mg/dL)	392	15.30 ± 3.92	16.35 ± 4.84	<0.001
AST (IU/L)	479	30.8 ± 19.7	28.8 ± 15.1	0.007
ALT (IU/L)	479	31.9 ± 28.2	26.8 ± 21.3	<0.001
GGT (IU/L)	451	66.1 ± 96.1	48.4 ± 69.6	<0.001
ALP (IU/L)	309	224.1 ± 87.9	184.7 ± 67.5	<0.001
CPK (mg/dL)	301	127.8 ± 82.9	132.0 ± 95.6	0.416
SBP (mmHg)	489	130.4 ± 13.5	128.6 ± 12.4	0.002
DBP (mmHg)	486	76.0 ± 9.5	74.8 ± 8.8	0.004
HbA1c (%)	308	6.46 ± 1.41	6.39 ± 1.29	0.187

Data are expressed as mean ± standard deviation safety parameters from baseline to LOCF. The paired *t* test was used to examine the significance of within-group changes. LOCF: last observation carried forward; AST: aspartate aminotransferase; ALT: alanine aminotransferase; GGT: gamma-glutamyl transpeptidase; ALP: alkaline phosphatase; CPK: creatine phosphokinase; SBP: systolic blood pressure; DBP: diastolic blood pressure; HbA1c: glycated hemoglobin.

### Efficacy assessments

**Figure 1:**
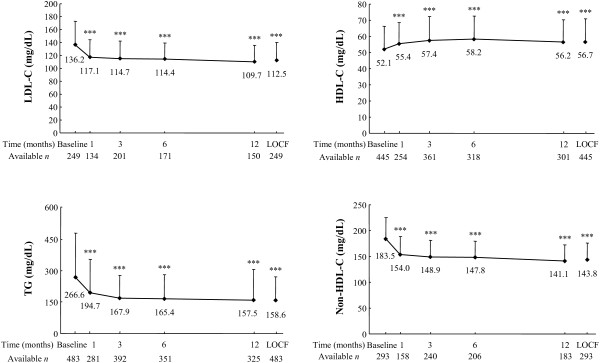
**Changes in LDL-C, HDL-C, TG, and non-HDL-C levels from baseline to the LOCF.** Data are expressed as mean ± standard deviation. The paired *t* test was used to examine the significance of within-group changes from baseline to the last observation carried forward (LOCF). *** p < 0.001. LDL-C levels were estimated using the Friedewald formula.

Efficacy was assessed in 622 patients. The changes in LDL-C, HDL-C, TG, and non-HDL-C from baseline to LOCF and to each time-point are shown in Figure 1. LDL-C decreased significantly by 17.4% from 136.2 ± 36.6 mg/dL at baseline to 112.5 ± 27.4 mg/dL at LOCF (change: –23.7 ± 36.1 mg/dL; p < 0.001). HDL-C increased by 8.8% from 52.1 ± 14.2 mg/dL to 56.7 ± 14.3 mg/dL (change: 4.6 ± 11.2 mg/dL; p < 0.001). TG decreased by 40.5% from 266.6 ± 210.1 mg/dL to 158.6 ± 112.2 mg/dL (change: –108.0 ± 181.3 mg/dL; p < 0.001). Non-HDL-C decreased significantly by 21.6% from 183.5 ± 41.8 mg/dL to 143.8 ± 32.4 mg/dL (change: –39.6 ± 40.9 mg/dL; p < 0.001). All four parameters improved significantly from baseline to each evaluation time-point.

### The subgroup analysis for drug administration

In total, 659 patients included in the safety analysis were divided into three groups in terms of background characteristics at baseline. Table 4 shows these baseline background factors. Of these patients, 174 (26.4%) were in the EZE + BEZA group, 257 (39.0%) in the BEZA + EZE group, and 228 (34.6%) in the BEZA & EZE group. The mean ages were 62.5 ± 12.9, 59.2 ± 12.0 and 61.2 ± 14.2 years in the EZE + BEZA, BEZA + EZE and BEZA & EZE groups, respectively. The complications, concurrent diseases, purpose of combination therapy and concomitant medications showed no imbalances among these three groups. ADRs were noted in 14 subjects (8.0%) in the EZE + BEZA group, 19 (7.4%) in the BEZA + EZE group and 9 (3.9%) in the BEZA & EZE group. Gallstones were noted in 2 subjects (1.1%) in the EZE + BEZA group, 1 (0.4%) in the BEZA + EZE group and 1 (0.4%) in the BEZA & EZE group. ADR incidences did not differ significantly among the three groups (P = 0.172).

**Table 4:** Baseline patient characteristics (patients grouped according to treatment order)

**Characteristics**	**EZE + BEZA**		**BEZA + EZE**		**BEZA & EZE**	
	**n**	**mean ± SD/%**	**n**	**mean ± SD/%**	**n**	**mean ± SD/%**
Age (years)	174	62.5 ± 12.9	257	59.2 ± 12.0	228	61.2 ± 14.2
Men (%)	94	54.0	173	67.3	117	51.3
With complications (%)	152	87.4	220	85.6	179	78.5
Concurrent disease						
Hypertension (%)	106	60.9	141	54.9	121	53.1
Diabetes (%)	61	35.1	100	38.9	69	30.3
Hepatic disease (%)	34	19.5	68	26.5	50	21.9
Cardiac disease (%)	8	4.6	19	7.4	19	8.3
Renal disease (%)	2	1.1	8	3.1	10	4.4
Purpose of combination therapy^a^						
Primary prevention (%)	152	87.4	245	95.3	207	90.8
Secondary prevention (%)	5	2.9	11	4.3	10	4.4
Unknown (%)	17	9.8	1	0.4	11	4.8
Concomitant medications						
Drugs for hypertension (%)	104	59.8	141	54.9	110	48.2
Drugs for diabetes (%)	44	25.3	72	28.0	47	20.6
Statin (%)	3	1.7	4	1.6	7	3.1

Data are expressed as mean ± standard deviation or n and percent.

^a^Defined according to the Guidelines for the Prevention of Atherosclerotic Cardiovascular Disease 2007 [9]. EZE + BEZA: patients who were already taking ezetimibe and then added bezafibrate at the start of the study; BEZA + EZE: patients who were already taking bezafibrate and then added ezetimibe at the start of the study; BEZA & EZE: patients who started bezafibrate and ezetimibe concurrently.

For the 622 patients included in the efficacy analysis, Table 5 shows the change and the rate of change in lipid levels from baseline to the LOCF in each group. The mean treatment durations were 370.5, 377.1, and 348.3 days in the EZE + BEZA, BEZA + EZE, and BEZA & EZE groups, respectively. As in the entire patient population, LDL-C, HDL-C, TG, and non-HDL-C values improved between baseline and the LOCF in all three treatment groups. Among baseline lipid parameters, LDL-C was high in the BEZA + EZE group, TG was high in the EZE + BEZA group, and LDL-C, TG and non-HDL-C were high in the BEZA & EZE group.

**Table 5:** Changes in lipid parameters in all patients and patients grouped according to treatment order

**Parameter**	**n**	**Baseline**	**LOCF**	**Change**	**%**	**p-value**
**LDL-C**						
Total	249	136.2 ± 36.6	112.5 ± 27.4	−23.7 ± 36.1	−17.4	<0.001
EZE + BEZA	67	120.0 ± 31.1	110.5 ± 22.9	−9.4 ± 31.7	−7.8	0.017
BEZA + EZE	99	143.6 ± 32.4	114.6 ± 25.1	−29.1 ± 29.0	−20.3	<0.001
BEZA & EZE	83	140.6 ± 41.5	111.6 ± 33.0	−29.0 ± 43.5	−20.6	<0.001
**HDL-C**						
Total	445	52.1 ± 14.2	56.7 ± 14.3	4.6 ± 11.2	8.8	<0.001
EZE + BEZA	138	52.5 ± 14.4	58.2 ± 14.8	5.7 ± 10.3	10.9	<0.001
BEZA + EZE	164	53.0 ± 15.2	55.4 ± 14.3	2.4 ± 12.0	4.5	0.012
BEZA & EZE	143	50.6 ± 12.6	56.9 ± 13.7	6.3 ± 10.9	12.5	<0.001
**TG**						
Total	483	266.6 ± 210.1	158.6 ± 112.2	−108.0 ± 181.3	−40.5	<0.001
EZE + BEZA	143	274.9 ± 169.0	147.6 ± 77.8	−127.3 ± 149.7	−46.3	<0.001
BEZA + EZE	183	244.0 ± 193.0	166.0 ± 125.0	−78.0 ± 193.7	−32.0	<0.001
BEZA & EZE	157	285.4 ± 256.7	160.1 ± 122.2	−125.2 ± 189.0	−43.9	<0.001
**Non-HDL-C**						
Total	293	183.5 ± 41.8	143.8 ± 32.4	−39.6 ± 40.9	−21.6	<0.001
EZE + BEZA	85	168.2 ± 33.2	142.7 ± 30.0	−25.5 ± 33.2	−15.2	<0.001
BEZA + EZE	112	187.3 ± 43.1	144.1 ± 29.2	−43.1 ± 39.2	−23.0	<0.001
BEZA & EZE	96	192.5 ± 43.7	144.5 ± 38.0	−48.0 ± 46.0	−24.9	<0.001

Data are expressed as mean ± standard deviation. “Change” indicates the change from baseline to the LOCF value. “%” indicates rate of change from baseline to the LOCF. The paired *t* test was used to examine the significance of within-group changes. LDL-C was estimated using the Friedewald formula. LOCF: last observation carried forward; EZE + BEZA: patients who were already taking ezetimibe and then added bezafibrate at the start of the study; BEZA + EZE: patients who were already taking bezafibrate and then added ezetimibe at the start of the study; BEZA & EZE: patients who started bezafibrate and ezetimibe concurrently.

The changes in LDL-C, HDL-C, TG and non-HDL-C from baseline to the LOCF were compared among the EZE + BEZA, BEZA + EZE and BEZA & EZE groups employing an ANCOVA model. Only changes in HDL-C and TG differed significantly among the three groups (LDL-C: P = 0.471, HDL-C: P = 0.008, TG: P = 0.039, non-HDL-C: P = 0.343). The adjusted change in HDL-C from baseline (estimate ± standard error) was 5.8 ± 0.9 mg/dL in the EZE + BEZA group, 2.6 ± 0.8 mg/dL in the BEZA + EZE group and 5.8 ± 0.9 mg/dL in the BEZA & EZE group, and the adjusted change in TG was −121.3 ± 8.1 mg/dL in the EZE + BEZA group, –94.4 ± 7.1 mg/dL in the BEZA + EZE group and −111.6 ± 7.7 mg/dL in the BEZA & EZE group.

## Discussion

Fibrates are thought to be the most effective drugs for treating low HDL-C and high TG levels [18,19]. Bezafibrate (400 mg/day) was reported to decrease TG levels by 45.4% and increase HDL-C levels by 14.0% in the J-BENEFIT study [20]. Concurrent administration of a fibrate and a statin might be a feasible treatment option for patients with mixed dyslipidemia who also have relatively high LDL-C levels. However, some caution is necessary because a high risk of rhabdomyolysis was reported during concurrent use of these drugs, especially in patients with renal dysfunction complications [21].

Ezetimibe acts by inhibiting the enterohepatic recirculation of lipids. In post-kidney transplantation patients, ezetimibe was found to stabilize creatinine clearance and also to suppress further decreases in renal function [22]. It differs from fibrates, which pass through the kidneys and are excreted in urine. A meta-analysis revealed that 10 mg/day ezetimibe as monotherapy decreased LDL-C by 18.58%, increased HDL-C by 3.00%, and decreased TG by 8.06% [23]. Therefore, bezafibrate in combination with ezetimibe is thought to be a feasible approach to reducing residual cardiovascular risk because this combination is expected to improve low HDL-C and high TG levels and to decrease LDL-C levels, while providing a good safety profile.

Fibrates reportedly increase the excretion of cholesterol into bile and may promote gallstone formation [24]. Ezetimibe does not promote cholelithiasis, according to one study [25]. To date, however, the risk of gallstones with bezafibrate in combination with ezetimibe has not been adequately evaluated. Therefore, in this study, we performed ultrasonography at baseline and at the end of the observation period to detect gallstones which had developed during combination therapy. We also evaluated the efficacy of this combination therapy in terms of improvements in lipid levels.

ADRs occurred in 6.4% of patients, which was similar to the value of 5.1% reported in our previous study (J-BENEFIT) in which bezafibrate was administered alone [20], and to the rate of 7.0% in patients treated with fenofibrate in combination with ezetimibe [13]. Severe ADRs occurred in three patients and the administration of these drugs was terminated in all three. None of the patients in this study experienced rhabdomyolysis.

All four patients with gallstones (0.6%) had diabetes in addition to dyslipidemia and were > 50 years old. Three of these patients were obese with BMIs of 28.6, 29.6, and 30.1 kg/m^2^. Because dyslipidemia, diabetes, advanced age, and obesity are known risk factors for gallstones [26], it seems likely that these subjects had physiological features that predisposed them to gallstone formation. The gallstone incidence based on the study duration was 1.1 per 100 person-years. The incidence of gallstones in the general population was reported to be 1.4 per 100 person-years [27] or 3.56%/year in an epidemiological study conducted in Taiwan that examined diabetic patients without gallstones [28]. Thus, the gallstone incidence in this study did not differ from that in the general population.

In terms of the impact of combination therapy on renal function, we observed a significant increase in creatinine levels between baseline and LOCF, although the magnitude of the increase was not clinically meaningful. However, because we did not determine the estimated glomerular filtration rate (eGFR), further studies will be necessary to evaluate the effects of this combination on renal function. We also considered the impact of combination therapy on liver functions. Co-administration of fenofibrate and ezetimibe reportedly increases the risk of hepatic dysfunction [13,15]. In our study, however, AST, ALT, GGT, and ALP decreased significantly from baseline to the LOCF. Bezafibrate accelerates fatty acid beta-oxidation by stimulating PPAR alpha receptors in the liver [29], and was reported to have lower hepatotoxicity than fenofibrate in mitochondrial toxicity studies using rat livers [30].

In the safety evaluation, co-administration of bezafibrate and ezetimibe was shown to not increase the risk of gallstone formation. In addition, liver function test values decreased, and BMI, systolic blood pressure, diastolic blood pressure and glycated hemoglobin levels showed no adverse influences. On the other hand, “increased blood CPK” and “myalgia” were noted as ADRs, and creatinine and blood urea nitrogen levels were significantly increased. As described in the exclusion criteria for this study, bezafibrate should not be administered to patients with a serum creatinine level ≥ 2.0 mg/dL.

In the efficacy analysis, LDL-C, TG, and non-HDL-C levels decreased and HDL-C increased significantly from baseline to the LOCF during combination therapy (all, p < 0.001). In particular, non-HDL-C levels decreased significantly from 183.5 mg/dL at baseline to 143.8 mg/dL at the LOCF (change: –39.6 mg/dL). This change is greater than that in the J-BENEFIT study [17], in which 2818 patients received bezafibrate monotherapy and non-HDL-C levels decreased from 178.3 mg/dL at baseline to 160.2 mg/dL at the LOCF (change: –18.1 mg/dL). These observations suggest that the combination therapy used in this study had beneficial effects on lipid profiles. The non-HDL-C fraction contains the lipoproteins that can cause arteriosclerosis, including remnant lipoproteins, and higher non-HDL-C levels were suggested to be associated with greater risk of coronary artery disease [31]. It was also reported that non-HDL-C is a better marker than LDL-C for estimating the risk of arteriosclerotic disease [32]. In particular, the Japan Atherosclerosis Society recommends that, in patients with TG > 400 mg/dL, non-HDL-C should be used rather than LDL-C as a clinical marker.

In the subgroup analysis, when the lipid parameter changes at the LOCF from the baseline level in each of the three groups (EZE + BEZA, BEZA + EZE and BEZA & EZE groups) were compared, significant improvement was seen in all lipid parameters. In addition, comparisons among the three groups revealed no significant differences in LDL-C or non-HDL-C. These efficacies were similar in the three groups and a complementary effect was seen. On the other hand, even after adjusting for baseline values, the analysis of covariance (ANCOVA) method showed significant differences in HDL-C and TG. These observations might be attributable to HDL-C and TG responding differently to the treatment interventions.

Our study has several important limitations. First, this was conducted as a post-marketing, prospective observational study, and was designed to examine and confirm the safety and efficacy of bezafibrate in combination with ezetimibe in patients with dyslipidemia, without a control group. Because it is impossible to remove all of the potential confounding factors, our results should be interpreted with caution until a randomized controlled study with a large number of patients can be performed. Second, for logistic, economic, and other reasons, laboratory parameters were measured at each of the participating institutions, rather than in a central laboratory. Third, LDL-C levels were estimated using the Friedewald formula. Therefore, cases with TG ≥ 400 mg were not evaluated for LDL-C [17]. Fourth, the usefulness of triple combination therapy with fenofibrate, ezetimibe and a statin has recently been reported [33,34]. Since this study was conducted for the purpose of clarifying the safety and efficacy of combination therapy with bezafibrate and ezetimibe, investigation of triple combination therapy with the addition of a statin was not part of the study design. Even in the case of combination therapy with bezafibrate and ezetimibe, an insufficient effect was seen in some patients. It is therefore necessary to sufficiently investigate triple combination therapy with the addition of a statin in future studies.

Nevertheless, this was the first study to examine the safety and efficacy of bezafibrate in combination with ezetimibe, and we confirmed that this combination achieved significant improvements in LDL-C, HDL-C, TG, and non-HDL-C levels. From the perspective of coronary artery disease prevention, this combination is useful as part of comprehensive management regimen for dyslipidemia.

## Conclusions

From the perspective of coronary artery disease prevention, comprehensive management of dyslipidemia is necessary by treating patients with low HDL-C and/or high TG levels in addition to those with high LDL-C levels. Based on our results, bezafibrate in combination with ezetimibe is a safe and effective treatment option that achieved marked improvements in LDL-C levels together with the expected increase in HDL-C levels and a decrease in TG levels.

## Abbreviations

ADRs: Adverse drug reactions; ALP: Alkaline phosphatase; ALT: Alanine aminotransferase; ANCOVA: Analysis of covariance; AST: Aspartate aminotransferase; BEZA & EZE: Patients who started bezafibrate and ezetimibe concurrently; BEZA + EZE: Patients who were already taking bezafibrate and then added ezetimibe at the start of the study; BMI: Body mass index; EZE + BEZA: Patients who were already taking ezetimibe and then added bezafibrate at the start of the study; GGT: Gamma-glutamyl transpeptidase; GPSP: Good Post-marketing Study Practice; HDL-C: High-density lipoprotein-cholesterol; LDL-C: Low density lipoprotein-cholesterol; LOCF: Last observation carried forward; PPAR: Peroxisome proliferator-activated receptor; SD: Standard deviation; TG: Triglyceride.

## Competing interests

This work was sponsored by KISSEI Pharmaceutical Co., Ltd., Japan. T. Teramoto has received research funds from Shionogi Co., Ltd., Astellas Pharma Inc., MSD K.K., and Daiichi-Sankyo Co., Ltd., and has received consulting fees, lecture fees, and/or honoraria from Astellas Pharma Inc., Takeda Pharmaceutical Co., Ltd., AstraZeneca K.K., MSD K.K., KISSEI Pharmaceutical Co., Ltd., Kowa Co., Ltd., Shionogi Co., Ltd., Daiichi-Sankyo Co., Ltd., Pfizer Inc., and Bayer Yakuhin, Ltd. K. Abe and T. Taneyama are employees of KISSEI Pharmaceutical Co., Ltd., the sponsor of this study.

## Authors’ contributions

KISSEI Pharmaceutical Co., Ltd. planned this study, helped develop the draft protocol, and performed the statistical analysis. T. Teramoto provided medical advice, reviewed the results, and compiled this paper. K. Abe and T. Taneyama are employees of KISSEI Pharmaceutical Co., Ltd. All authors read and approved the final manuscript.
